# Prostate cancer and therapeutic challenges

**DOI:** 10.1186/s40709-020-00128-z

**Published:** 2020-12-10

**Authors:** Yousef MohammedRabaa Hawsawi, Samar Abdullah Zailaie, Atif Abdulwahab A. Oyouni, Othman Rashed Alzahrani, Osama Mohamed Alamer, Saad Ali S. Aljohani

**Affiliations:** 1 Saudi Human Genome Program-Jeddah Satellite Laboratory, Research Center, King Faisal Specialist Hospital and Research Center, MBC-J04, P.O. Box 40047, Jeddah, 21499 Kingdom of Saudi Arabia; 2 College of Medicine, Al-Faisal University, P.O. Box 50927, Riyadh, 11533 Saudi Arabia; 3 Department of Biology, Faculty of Sciences, University of Tabuk, Tabuk, Kingdom of Saudi Arabia; 4 Department of Medical Laboratory Technology, Faculty of Applied Medical Science, University of Tabuk, Tabuk, Kingdom of Saudi Arabia; 5 Department of Basic Medical Sciences, Faculty of Medicine, Al-Rayan Colleges, Almadinah Almunawarah, Kingdom of Saudi Arabia

**Keywords:** Prostate cancer, Androgens, Androgen receptor, Anti-androgen therapy, Heat-shock protein

## Abstract

Prostate cancer (PC) is the most prevalent type of cancer in men worldwide. In Saudi Arabia, the rate of PC is increasing annually. The sex steroid hormones androgens and their receptors have critical roles in PC development and progression. Additionally, apoptosis-related proteins such as heat-shock proteins are vital molecules in PC development. Steroid hormone-deprivation therapies remain the essential treatment for patients with metastatic PCs; however, acquired resistance to hormone deprivation and the transition to PC androgen independence is a major health obstacle. In this review, we aim to detail the roles of androgens, androgen receptors and sex steroid hormones in inducing apoptosis in PC.

## Introduction

Prostate cancer (PC) is becoming the highest rate of cancer of all types in men in the United States (US), even though PC molecular biology is well understood. In a 2015 study, nearly 220,800 new cases of PC were reported there with 27,540 deaths [1]. In Saudi Arabia, PC has been ranked as the fifth most commonly diagnosed cancer among Saudi men with 324 newly diagnosed cases in 2014 [2]. In clinical research and for healthcare systems worldwide, these figures present a major challenge.

Androgens have critical roles in PC development and progression in men as PC cells are androgen receptor (AR)-dependent for development. The majority of PCs cases are hormone dependent; therefore, anti-hormone therapies have had a profound impact in reducing the burden of this disease worldwide.

Distinct parameters in PC such as tumour volume and pathological grade show a strong correlation with malignancy [3]. High percentages of AR-positive cells present a strong correlation with an excellent prognosis in PC [4]. AR-negative PC is the least common subtype; hence, only scant attention has been paid to it.

Usually, a choice of therapy is not offered to individuals diagnosed with PC since it is an indolent disease with low PC-specific mortality rates and in which co-morbidities apparently are the cause of death [5, 6]. In cases of aggressive PC, however, adjuvant radiotherapy and surgery are the main therapies of choice. If the disease progresses despite these primary treatments or has reached advanced stages (metastasized), only then is hormone therapy utilized.

Although androgen-deprivation therapy (ADT) remains the normal treatment for PC, a significant number of affected individuals develop castration-resistant PC (CRPC) in the long term. CRPC is the progression of PC after the administration of ADT for 2 to 3 years [7, 8]. The main clinical characteristics of CRPC are increasing levels of prostate-specific antigen (PSA), the progression of PC, and metastases to nearby tissue, specifically bone. The AR and its signalling pathways play a critical role in prostate gland haemostasis. Additionally, the AR has an indispensable role in the acquired resistance mechanism of PC. The distinct domains in ARs have long been thought to contribute to PC resistance mechanisms.

## Androgen receptor

The AR is a 110 kb ligand-induced transcription factor that belongs to the steroid hormone group of the nuclear receptor superfamily [9]. The gene for AR is located on the X chromosome at the Xq11-12 site and codes for a protein that contains three major functional domains: the N-terminal domain (NTD), DNA-binding domain (DBD), and ligand binding region (LBD) [9]. The AR is mainly located in the cytoplasm and is associated with chaperone proteins such as heat-shock proteins (HSPs) [9]. In men, the primary function of the AR is sexual development and differentiation in addition to the normal functions of the prostate gland. The activation of the AR is initiated by the binding of steroid hormones—mainly 5α-dihydrotestosterone (DHT) and testosterone—to the AR. Notably, the AR has a higher affinity for DHT than for testosterone [9]. Upon ligand binding, the AR-chaperone complex is released and allows the translocation of the AR to the nucleus where it dimerizes and binds to the AR-response elements (AREs) of target genes. ARE–androgen complexes modulate gene transcription for hundreds of target genes involved in cell cycle control and apoptosis in prostate epithelial cells as well as for genes implicated in the normal functioning of the male reproductive system [10, 11].

Defects in AR signalling pathways play an important role in carcinogenesis of the prostate gland. Several studies have identified changes in AR signalling pathways that contribute to PC such as somatic mutations of the AR [12, 13], the overexpression of the AR, and changes in androgen metabolism [14, 15]. The function of the AR in men is similar to the function of estrogen receptor in women in terms of inducing apoptosis [16, 17].

## The role of the androgen receptor in prostate cancer

PC cells are regulated by sex steroid hormones. Androgen deprivation leads to hypersensitivity to androgens in Lymph Node Carcinoma of the Prostate (LNCaP) PC cells [8, 18], and long-term androgen deprivation further leads to androgen-induced apoptosis [7, 19]. In many cases, PC initiation can be assigned to distinct growth-promoting pathway activation such as the androgen-dependent upregulation of the E-twenty-six (ETS) family members of transcription factors. The promoter of the AR regulates the TMPRSS2 gene in the coding region of ETS family members erythroblast transformation-specific (ERG) and ETS variant 1 (ETV1) [20–22]. It has been reported that this fusion induction is dependent on three different enzymes that cause chromosomal translocation: activation-induced cytidine deaminase (AID), LINE-1 repeat-encoded ORF2 endonuclease, and topoisomerase II beta (TOP2B) [23, 24]. The dysregulation of other signaling pathways—including the PI3K and RAS/RAF pathways—has been reported to have a role in the initiation and progression of PC that has been confirmed through their genomic profiling [25, 26]. The AR, nonetheless, remains the master regulator as a signaling pathway for PC initiation and progression more than any AR-independent pathway does. Therefore, androgen suppression/deprivation in combination with other therapies has become the milestone for PC treatment.

## History of hormonal therapies for prostate cancer

In the 1940s, death occurred in most PC patients diagnosed at an advanced stage of the disease with survival rates of 1 to 2 years after diagnosis. During this time, the influence of ADT on metastatic PC (MPC) was reported by Charles Huggins using the synthetic estrogen diethylstilbestrol (DES) or surgical castration [19]. Following Huggins’s discovery, ADT was utilized as a gold standard for the treatment of MPC heralding a new era for the development of novel therapeutic agents.

In the late 1960s, the discovery of the AR by three independent groups—Anderson and Liao [27], Bruchovsky and Wilson [28], and Mainwaring [29]—initiated the search for androgen antagonists. Flutamide, the first non-steroidal anti-androgen, was discovered in the mid-1970s [30] and was approved for the treatment of PC by the US Food and Drug Administration (FDA) in 1989. After this finding, randomized trials were run using other non-steroidal anti-androgens including bicalutamide and nilutamide, and the results were compared with those using castration in MPC patients. The results indicated better tolerance of anti-androgen drugs compared with castration; however, they proved to be inferior therapies with regard to overall survival (OS) and progression-free survival (PFS) [31, 32]. Slightly longer PFS was reported by Crawford and colleagues [33] when flutamide and leuprolide were combined. As a result, a combined androgen blockade was a preferable option for physicians in the US and was used as the initial therapy for advanced PC. Soon after, Laufer and co-authors used androgen deprivation in various combinations in a randomized phase III trial [34], but only three showed a noteworthy, measurable benefit for a complete androgen blockade.

Between the 1970s and 1980s, new approaches were also developed for hormone treatments to either block the production of adrenal androgen or inhibit androgen activity in the AR. In 1971, the structure of the hypothalamic hormone known as the luteinizing hormone (LH)-releasing hormone (LHRH) called the gonadotropin-releasing hormone (GnRH)) was described by Andrew Schally and Roger Guillemin [35]. Using daily doses of LHRH agonists to treat advanced PC patients demonstrated a 75% decrease in serum testosterone levels, a decrease or normalization of plasma acid phosphatase levels, and, most importantly, a significant decrease in cancer-associated bone pain [36]. Subsequently, many synthetic LHRH agonists such as buserelin, leuprolide, nafarelin, and goserelin, were developed for clinical use [37]. Furthermore, LHRH antagonists such as abarelix, cetrorelix, and orgalutran were developed and tested for treating advanced PC in men [37]. While GnRH agonists (such as leuprolide and goserelin) clearly act to interrupt the physiological stimulation of the GnRH receptor that in turn decreases its sensitivity, their antagonists (such as degarelix) directly block GnRH stimulation [38].

## Hormone-resistant PC therapies

Targeting new PC cell populations after long-term sex steroid deprivation leads to resistance to anti-hormone therapy. These cells are characterized by sex hormone-independent growth; however, vulnerability may present in the form of sex steroid-induced apoptosis [7, 8, 39]. For prostate hormone refractory cancer, cytotoxic chemotherapy is still regarded as a therapeutic choice. At present, it is believed that in hormone therapy-resistant PC, the AR is still functional and can be abrogated to stop progression. At the same time, cytotoxic chemotherapy is utilized as well.

Castration resistance mechanisms remain unclear, but there are four main suggested ones for CRPC development under androgen deprivation. The first mechanism illustrated that PC cells became more sensitive with decrease in the androgen threshold although androgen was sufficiently overexpressed to activate the AR because of in situ intra-tumour [40] and adrenal gland synthesis [41]. This was also accompanied by a reduction of the CYP3A4, CYP3A5, and CYP3A7 androgen-inactivating enzyme levels in PC patient tissue samples [42]. The second mechanism reports AR gene mutations that in turn substitute amino acids in the LBD proteins and decrease its specificity and selectivity. These mutant proteins in the AR lead to non-specific binding with other steroidal hormones (glucocorticoids, progesterone, and estrogen) that increases AR transcriptional activity resulting in the growth of prostate cancer [43]. Another mechanism that has a role in CRPC development is a ligand-independent AR activation in which the AR is phosphorylated through crosstalk with different kinase enzymes (Akt, HER2, and Ack1) and a long, non-coding RNA (PCGEM1) binds to the AR to encourage its target gene transcription [15]. The fourth mechanism is AR-independent in which dead PC cells produce proinflammation that causes B and T cell infiltration. B cell infiltration produces lymphotoxin and other factors that in turn increase the signalling of Stat3 that is essential for PC cell survival in a hormone-free pathway [44]. In addition, Bcl-2 (anti-apoptotic) protein upregulation protects cancer cells from castration-induced apoptosis [45]. Moreover, glucocorticoid receptor overexpression has a role in the survival of cancer cells [46].

The antimitotic chemotherapeutic agent docetaxel binds the β-subunits of tubulin in microtubules [47] inducing apoptosis by inhibiting spindle fibre assembly. In CRPC, docetaxel phosphorylates Bcl-2 and thus activates caspases and triggers apoptosis in vivo and in vitro [48]. Additionally, docetaxel upregulates p53 and activates the proteinase-activated receptor 1 (PAR-1) that plays a critical role in triggering apoptosis pathways [49]. After several clinical trials, in 2004 the US FDA approved the use of docetaxel-based chemotherapy which significantly palliates cancer-associated symptoms and prolongs the OS of patients with metastatic castration-resistant PC (mCRPC) [50]. Subsequently, De Bono and co-authors compared cabazitaxel with mitoxantrone (topoisomerase type II inhibitor) in mCRPC patients previously treated with docetaxel in a trial comparing cabazitaxel plus prednisone with mitoxantrone plus prednisone in hormone-refractory metastatic PC (TROPIC trial). In the cabazitaxel group, mortality was significantly decreased. Later, De Bono and colleagues reported the ability of abiraterone acetate to inhibit androgen biosynthesis that in turn prolonged the OS of mCRPC patients who had previously received chemotherapy [51]. It is known that abiraterone acetate acts as a Cytochrome P450 (CYP17) inhibitor in which CYP17 plays a vital role in androgen production from estrogens and glucocorticoids through the adrenal steroid hormone synthetic pathway [20]. Galeterone, another steroidal CYP17 inhibitor, has also been shown to have promising anticancer properties. Indeed, galeterone was approved by the FDA for a phase I trial in 2015. The mechanism by which galeterone induces tumour apoptosis is not fully understood, but it has been shown to act as a multi-targeting agent disrupting AR signalling.

A clinical trial was conducted by Scher and colleagues [22] to assess the anti-tumour activity and safety of MDV3100 (or enzalutamide, an AR antagonist that inhibits androgen-AR binding) in men with CRPC. The authors reported their anti-tumour effects at different doses by reducing serum PSA levels and stabilizing bone density in 56% of patients. Another study reported a decrease in the mortality of patients by 76% when using enzalutamide with a median PFS of 19.4 months, whereas a median PFS of only 5.7 months was recorded for the bicalutamide administrated group in the PC (STRIVE) Trial [21].

ARN-509 is an example of a potent, competitive, and purely antagonistic anti-androgen that has been assessed in phase I/II trials in CRPC patients. EPI-001 is a novel, small peptide that acts directly on the N-terminal domain of the AR. AZD3514 is a novel, selective AR downregulating drug (SARD) [24]. Additionally, Smith and colleagues [23] started a new clinical trial to evaluate cabozantinib (XL184), an orally bioavailable tyrosine kinase inhibitor that has an opposite action against the hepatocyte growth factor receptor MET and vascular endothelial growth factor receptor 2 (VEGFR2) in CRPC patients.

ODM-201 (darolutamide, a novel second-generation nonsteroidal anti-androgen) has also been reported by Moilanen et al. as a novel synthetic anti-androgen compound that is unlike known anti-androgens like enzalutamide [25]. Moilanen et al. have also reported the anti-proliferative effect of ODM-201 in tumour growth using castration-resistant VCaP xenograft as a study model in vivo [24].

## Sex steroid induced apoptosis in hormone-resistant PC

After 2 to 3 years of ADT, PC can, despite continuous hormonal manipulation, progress to form the CRPC phenotype. CRPC is usually treated using anti-androgens that directly block the action of the AR. Several variants of LNCaP PC cell lines have been used in vitro to mimic anti-androgen resistance in PC and to demonstrate the biological alterations that occur. In 2011, studies [52] demonstrated the blockade of the cell cycle in G1 by the regulation of c-Myc, Skp2, and p27^kip^ via the AR in variants of LNCaP PC cell lines. Furthermore, they found an increase in growth inhibition of relapsed tumours when higher doses of testosterone were used, indicating that the manipulation of the androgen/AR signalling pathway in AR-positive metastatic PC may be a potential therapy target.

In 2010, Kawata and colleagues [18] reported that extended treatment with bicalutamide on a bicalutamide-resistant subline (LNCaP-BC2) resulted in the overexpression of the AR and hypersensitivity to low levels of androgen. The authors proposed the overexpression of phosphorylated AR (pAR^210^) and androgen hypersensitivity as a possible mechanism; however, LNCaP PC cells became a population susceptible to androgen-induced apoptosis after prolonged androgen deprivation [52]. Currently, androgen-induced apoptosis in CRPC is clinically proven. Akakura and colleagues [53] used intermittent anti-androgen therapy (IAT) to demonstrate the inhibition of anti-androgen resistant PC by androgen action. Recently, Schweizer and co-authors [54] found that monitoring either PSA levels or radiologically identified CRPC results in a 50% response rate to androgen therapy.

## Targeting of heat-shock proteins in PC

More attention has been given to the use of heat-shock proteins (HSPs) as therapeutic targets in PC, recently. HSPs are highly conserved proteins and are found in most organisms, synthesized in response to a variety of chemical and physiological stresses [55].. HSPs are classified into different families according to their molecular weight including small Hsp, Hsp70, Hsp60, Hsp90, and Hsp100 [55]. HSPs act as chaperones to other cellular proteins and play an essential role in protecting cells from the harmful consequences of stress by maintaining protein stability and controlling protein function [30]. Under normal conditions, HSPs are expressed at a low level; however, in different types of cancer, including PC, they have been shown to be overexpressed [31]. HSPs are also involved in cancer progression, metastasis, and anti-cancer drug resistance [32].

Natural, product-based compounds were the first to be used to target HSPs. In 1994, Geldanamycin (GA, benzoquinone ansamycin) was the first Hsp90 inhibitor identified [56]. This study discovered the binding of GA to Hsp90 which interferes with its functions. Shortly thereafter, Itoh and his colleagues recognized a drug named cisplatin as an HSP inhibitor that reduces Hsp90 chaperone activity [57]. GA was not, however, pursued in clinical trials due its significant toxicity on cells as well as to the difficulty in producing it. Additional first-generation Hsp90 inhibitors derived from GA (with lower cell toxicity) have been originated by structurally modifying GA, including 17-Allylamino-17-demethoxygeldanamycin (17-AAG) and 17-dimethylaminoethylamino-17-demethoxygeldanamycin (17-DMAG). Although both drugs entered clinical trials, the trials were terminated because of the drugs’ toxicity profiles in patients [36].

Structural studies have also established purine-based inhibitors that have a higher specificity for Hsp90 [58, 59]. Among these inhibitors, PU-H71 (8-[(6-iodo-1,3-benzodioxol-5-yl)sulfanyl]-9-[3-(propan-2-ylamino) propyl]purin-6-amine) showed a promising anti-cancer result in a breast cancer cell line [38], in non-Hodgkin’s lymphoma [60], and in hepatocellular carcinoma [41].

Pifithrin-µ (PFT-μ) (2-phenylethynesulfonamide) is a small molecule that directly binds to Hsp70 and inhibits its function [42]. In vivo and in vitro studies conducted by Sekihara and his group indicated that PFT-µ induced cell death in LNCaP suggesting it might be a promising agent to treat PC [43]. The same group has also revealed that the compensation of hyperthermia with PFT-μ in three human PC cell lines (LNCaP, PC-3, and DU-145) induced the apoptosis pathway and decreased proliferating cancer cells.

Second-generation Hsp90 inhibitors such as onalespib (AT13387) inhibited cell proliferation in the androgen-dependent PC cell lines VCaP and 22Rv1 as well as in the androgen-independent cell line LNCaP95 [44]. Additionally, XL888, a novel, synthetic, small molecule targeting Hsp90 has also induced apoptosis in PC cell lines when combined with other Hsp90 inhibitors [61].

Targeting HSPs is, therefore, a promising future direction for researchers aiming to design and synthesize high efficacy, low toxicity inhibitors that will provide novel approaches for treating neoplastic diseases [61], including PC.

## Applied bioinformatics and prostate cancer

Today, bioinformatics is used in identifying and validating drug targets and in developing biomarkers to increase their therapeutic benefits. Recently, this has become more applicable due to the availability of cellular signalling pathway data and to the development of integrated computational and experimental projects. Promising future applications include the following: the development of RNA-based biosensors that can be integrated into a cancer diagnostic device; the programmed engineering of bacteria that can target tumour and in situ release a therapeutic agent; the use of viral vectors as tumour recognizing tools in gene therapy; and the production of complex chemotherapeutic agents on a large scale [46]. Genetically manipulated bacteria to use in the development of treatment strategies in which vectors and expressed anti-tumour proteins could infect cancer cells [60] has become relatively easy. Recently, several bacterial strategies have been implemented in vivo including testing *Salmonella* sp. on different cancer cells, including PC. In addition, Alinezhad et al. [61] were able to validate novel biomarkers including gene silencing PLA2G7, RHOU, ACSM1, LAMB1 and CACNA1D, that resulted in the reduction of tumour-cell invasion in PC3 organoid cultures in combinations of clinical, functional, and bioinformatics studies.

## Conclusions

In this review, we provide an overview of PC and discuss the main role of the AR in its initiation and progression. The AR remains the master regulator of PC, but its deprivation can also lead to anti-hormonal therapy resistance. This highlights the need for clinical studies that focus on hormonal-resistant PC therapies and that target HSPs. In addition, various applications of bioinformatic tools and analyses could be good alternatives for designing PC therapeutics. The importance and relevance of this study is to amplify the response rate to long-term adjuvant anti-hormone therapy and to offer new strategies to target PC and reduce its high rate of mortality worldwide.

## Data Availability

Not applicable for this section.
